# MRI-based multivariate gray matter volumetric distance for predicting motor symptom progression in Parkinson's disease

**DOI:** 10.1038/s41598-023-44322-0

**Published:** 2023-10-17

**Authors:** Anupa A. Vijayakumari, Hubert H. Fernandez, Benjamin L. Walter

**Affiliations:** Center for Neurological Restoration, Cleveland Clinic, 9500 Euclid Avenue, Mail Code: S20, Cleveland, OH 44195 USA

**Keywords:** Predictive markers, Parkinson's disease, Parkinson's disease

## Abstract

While Parkinson's disease (PD)-related neurodegeneration is associated with structural changes in the brain, conventional magnetic resonance imaging (MRI) has proven less effective for clinical diagnosis due to its inability to reliably identify subtle changes early in the disease course. In this study, we aimed to develop a structural MRI-based biomarker to predict the rate of progression of motor symptoms in the early stages of PD. The study included 88 patients with PD and 120 healthy controls from the Parkinson's Progression Markers Initiative database; MRI at baseline and motor symptom scores assessed using the MDS-UPDRS-III at two time points (baseline and 48 months) were selected. Group-level volumetric analyses at baseline were not associated with the decline in motor functioning. Then, we developed a patient-specific multivariate gray matter volumetric distance and demonstrated that it could significantly predict changes in motor symptom scores (*P* < 0.05). Further, we classified patients as relatively slower and faster progressors with 89% accuracy using a support vector machine classifier. Thus, we identified a promising structural MRI-based biomarker for predicting the rate of progression of motor symptoms and classifying patients based on motor symptom severity.

## Introduction

Parkinson’s disease (PD) is a chronic, progressive neurodegenerative disease clinically diagnosed by cardinal motor symptoms such as bradykinesia, rigidity, tremor, gait and postural disturbances^1^. One of the important foci of current PD research is to identify robust biomarkers to predict the progression of motor symptoms and to diagnose PD based on symptom severity in the early stages, that is, when signs are subtle and non-diagnostic. Although different approaches to identifying biomarkers in PD have been attempted, ^2–6^ “gold standard” biomarkers that can be recommended for the clinical management of patients are still unavailable.

The hallmark pathophysiology of PD is the degeneration of the dopaminergic neurons in the substantia nigra leading to functional and structural alterations in the basal ganglia-thalamocortical circuits^7,8^ and the presence of Lewy bodies in several regions of the brain^9,10^. Atrophy in brain regions can be gauged macroscopically using structural magnetic resonance imaging (MRI). Although routine clinical MRI is considered “normal” in PD, voxel-based or surface-based morphometric (MRI-based techniques to detect subtle changes in the brain) studies have reported gray matter volume (GMV) loss in cortical and subcortical structures^11,12^, which were also correlated to the motor dysfunctions in PD^13,14^. MRI-based biomarkers could be a compelling option in a clinical setting since MRI is widely available, non-invasive, has standardized acquisition parameters, and can be seamlessly integrated into their clinical workflow. A recently identified MRI prognostic biomarker, i.e., a PD-related atrophy score, is promising but has limited accuracy in predicting outcomes in single patients with PD^4^. Therefore, there is an unmet demand to identify a robust imaging biomarker to monitor disease progression.

Previous brain morphometric studies have enhanced our understanding of the structural abnormalities and their relationship to motor symptoms in PD^11–14^. However, firm conclusions cannot be drawn from a clinician’s point of view. Clinical relevance can be achieved only when we address the critical involvement of brain region(s) with disease in individual patients. This has been very challenging, as the findings from previous studies carry information based on group-level analysis (i.e., comparing a group of patients with healthy controls) and are not specific to individual patients. Furthermore, heterogeneous cortical atrophy patterns were noted in the early stages of PD^15,16^. In such cases, if different brain regions are involved in the PD pathology in different patients, then the conventional group-level results can lead to inconclusive findings. Therefore, developing a method that summarizes the heterogeneity in multiple gray matter regions into a single score in individual patients would be beneficial for clinical decision-making.

In this study, we aimed to develop a structural MRI-based biomarker based on a patient-specific summary score of GMV heterogeneity of multiple brain regions using Mahalanobis distance (MD) (referred to as multivariate gray matter volumetric distance) to predict the long-term changes in motor symptoms. Another promising approach for biomarker discovery is to make predictions at the individual patient level. Therefore, we also made an attempt to classify PD patients based on the severity of motor symptoms using a machine learning technique. To do so, initially, we aimed to examine the association of GMV with the changes in motor symptoms in PD at a group-level. Then, we created a patient-specific imaging biomarker, a summary score created from multiple brain regions, and investigated its ability to predict the rate of progression of motor symptoms. Finally, we explored the ability of the imaging biomarker to classify patients based on the severity of motor symptoms using machine learning.

## Methods

### Participants

Data used in this study was downloaded from the Parkinson’s Progression Markers Initiative (PPMI) database (https://www.ppmi-info.org/access-data-specimens/download-data), RRID: SCR_006431 through a standard application process. For up-to-date information on the study, please visit www.ppmi-info.org. Inclusion criteria of the patients were (i) asymmetric resting tremor or asymmetric bradykinesia or two of resting tremor, bradykinesia, and rigidity; (ii) evidence of a positive DaTscan confirming the PD diagnosis; and (iii) patients who had not been treated within six months of enrollment. Exclusion criteria for all participants were: (i) a diagnosis of dementia or atypical PD syndromes; (ii) significant neurologic or psychiatric conditions; (iii) the presence of MRI motion artifacts, field distortions, intensity inhomogeneities, or detectable brain injuries. Motor symptoms were assessed using Part III of the Movement Disorder Society-Sponsored Revision of the Unified Parkinson's Disease Rating Scale (MDS-UPDRS-III)^17^. The total as well as the subscores of MDS-UPDRS-III, including rigidity (item 3.3), bradykinesia (items 3.4–3.8), postural instability and gait (items 3.10–3.13), postural-kinetic tremor (items 3.15–3.16), and resting tremor (item 3.17), were computed. For this study, we selected MDS-UPDRS-III scores at baseline and OFF-medication (to minimize the influence of PD (dopaminergic) medications) scores at 48 months (48 M) (due to the high availability of OFF-medication MDS-UPDRS-III data when compared to other time points) for predicting the long-term changes in the motor symptoms. Thus, a total of 88 patients with PD were selected for the study, and the flowchart of patient selection is illustrated in Supplementary Fig. S1 online. We also downloaded 130 age- and gender-matched health controls (HC) from the PPMI database. Among them, 10 HC were excluded due to MRI-related data quality issues. Thus, the final analysis included a total of 208 participants (N_HC_ = 120, N_PD_ = 88).

### Ethical approval

The PPMI study, which adhered to the Declaration of Helsinki and Good Clinical Practice guidelines^18^, has been registered on ClinicalTrials.gov with the identifier NCT01141023. PPMI participants provided written, informed consent to participate after the approval of the local ethics committees of the participating sites. A list of participant sites can be found at https://www.ppmi-info.org/about-ppmi/ppmi-clinical-sites. One of the authors (AAV) obtained permission to use PPMI data for this study, and all MRI as well as clinical data used for this study were downloaded in a de-identified format. The PPMI Data and Publications Committee reviewed our manuscript for administrative approval in accordance with PPMI policies. Therefore, the analyses presented in this article were conducted in accordance with approved PPMI guidelines.

### MRI data acquisition and processing

PPMI MRI images were acquired with standardized acquisition parameters on MRI scanners from different vendors (Siemens, Philips, and GE). We selected the T1-weighted (T1W) images from the participants scanned on a 3.0 T MRI scanner for this study. A description of scanning parameters is shown in Supplementary Table S1 online.

Pre-processing of the T1W images was carried out using Computational Anatomy Toolbox 12 (CAT12.8 (r1900); http://www.neuro.uni-jena.de/cat/) within SPM12 (Wellcome Department of Imaging Neuroscience Group, London, UK; http://www.fil.ion.ucl.ac.uk/spm) in MATLAB software (The Mathworks Inc.; MA, USA, R2021a). Briefly, after correcting for bias field inhomogeneity, the T1W images were segmented into gray matter (GM), white matter (WM), and cerebrospinal fluid (CSF)^19^. Then, GM images were normalized into the standard Montreal Neurological Institute (MNI) space using Diffeomorphic Anatomic Registration Through Exponentiated Lie algebra algorithm (DARTEL)^20^. Finally, smoothing was applied to the normalized GM images with a Gaussian kernel of a full width at half maximum (FWHM) of 8 mm. For all participants, segmented images were visually inspected, and image quality ratings (IQR: a combination of measurements of noise and spatial resolution) were above the “satisfactory” threshold (i.e., 75%). Using the “Estimate mean values inside ROI” function in CAT12, GMV was extracted from 40 regions of interest (ROIs) expected to be related to motor functions (Supplementary Table S2 online) from the automated anatomical labeling atlas 3 (AAL3)^21^. The total intracranial volume (TIV) for each participant was extracted using the “Estimate TIV” function (TIV = GM + WM + CSF). To control for inter-subject variations in head size, we divided GMV by the TIV of each participant to obtain normalized GMV (GMV_N_).

### MRI data harmonization

Since the MRI data was acquired on different scanners, the NeuroHarmonize software package^22^ was applied to remove the variability between scanners. NeuroHarmonize is an extension of the ComBat framework^23^, which uses an empirical Bayes approach to correct the effects of sites while preserving the biological variance in the data. In the present study, diagnosis, age, and sex were considered biological variables. The GMV_N_ generated from the 40 ROIs was harmonized using the publicly available Python package NeuroHarmonize (https://github.com/rpomponio/neuroHarmonize).

### Group-level gray matter volumetric analyses

In patients with PD, the association of harmonized GMV_N_ of motor-specific ROIs (n = 40) to the changes in motor outcome (MDS-UPDRS III: total and its subscores) was computed using partial correlation analysis. This analysis was performed while taking into account potential confounding factors such as age, gender, and disease duration. We considered a *P*-value threshold of < 0.001 (0.05 divided by 40) as statistically significant, accounting for multiple comparisons using the Bonferroni correction.

### Patient-specific multivariate gray matter volumetric distance

M_GMV_, multivariate gray matter volumetric distance was computed using MD^24^_._ MD is a generalization of the z-score in a multivariate space, which calculates a patient-specific distance from the HC distribution. Age was regressed out as a covariate using the fitlm method in MATLAB, and the residuals (GMV_N_^r^) were used to compute MD as follows:

$$ {\text{M}}_{{{\text{GMV}}}} = { }\sqrt {\left( {{\varvec{s}} - {\upmu }} \right)^{{\tau { }}} \cdot C^{ - 1} \cdot \left( {{\varvec{s}} - {\upmu }} \right)} $$

where s represents a vector of GMV_N_^r^ observations in each ROI in a single participant, µ is the vector of average GMV_N_^r^ of each ROI calculated from the HC, and C is the covariance matrix between brain regions (40 ROIs) across the HC. To reliably estimate the inverse covariance matrix $\left( {C^{ - 1} } \right)$, 100 HC were randomly selected and permuted 1000 times. The M_GMV_ of patients was calculated individually relative to each of the 1000 HC distributions, and the median values were reported.

### Statistical analysis

Analyses of demographic and clinical characteristics were performed using two-tailed *t*-tests or Chi-square tests, as appropriate. Assumptions for normality were tested for variables using the Shapiro–Wilk test. Changes in motor outcome were calculated by subtracting MDS-UPDRS-III scores at baseline from 48 M (∆ (MDS-UPDRS-III) = MDS-UPDRS-III_48M_—MDS-UPDRS-III_Baseline_). Positive change scores denote a decline in motor functioning. In a similar fashion, we also calculated the changes in subscores of MDS-UPDRS-III such as rigidity, bradykinesia, gait and postural instability, postural-kinetic tremor, and resting tremor^17^. For instance, rigidity change scores (Δ rigidity) were calculated by subtracting each patient's baseline rigidity subscore (item 3.3 on MDS-UPDRS-III) from their 48-month rigidity subscore. We tested whether M_GMV_ could predict changes in motor outcome using multiple linear regression models covaried for age and sex. The effect size of the regression model was reported using Cohen’s f^2^ ^25^. All analyses were performed using R software (version 4.1.2, https://www.r-project.org/). We applied the Bonferroni correction to account for multiple comparisons, encompassing both the overall motor changes and the five subscores. To achieve statistical significance, a threshold of *P* < 0.008 (0.05 divided by 6) was considered.

### Supervised machine learning for classification

We observed that M_GMV_ could predict the motor progression in patients with PD (detailed in the results section). So, we explored if we could develop a machine learning model capable of classifying patients based on the rate of progression of their motor symptoms. We categorized the patients into two groups, "slower progressors" (SP) and "faster progressors" (FP), based on the median value of ∆ (MDS-UPDRS-III) = 9. Horvath et al. defined a minimal clinically important difference threshold of 4.63 for worsening of motor symptoms over a median interval of 6 months^26^. Thus, the median value of 9 in this fairly early stage population may also be clinically meaningful. The Scikit-learn package of Python was used to develop the classifier^27^. To classify the patients, we used a support vector machine (SVM) classifier with a radial basis function kernel. The features used for developing the SVM classifier were M_GMV_, age, gender, and MDS-UPDRS-III at baseline; these features were standardized using StandardScalar, SKLearn (a ML library for Python), by removing the mean and scaling to unit variance. Among the eighty eight patients, forty six patients had a ∆ (MDS-UPDRS-III) ≤ 9 and were labeled as “0,” representing SP, and the rest (n = 42) with a ∆ (MDS-UPDRS-III) > 9 were labeled as “1,” representing FP. The data was split into training (70%, n = 61) and testing (30%, n = 27) datasets using the train/test split function in SKLearn. The testing dataset (n = 27) was held back and not used until the SVM classifier was completely trained using the training set (n = 61). The SVM classifier was fine-tuned by implementing GridSearchCV (a function in SKLearn for finding the optimal values for the hyperparameters of the classifier). To select the best set of parameters for the classification, the SVM classifier was trained with a ten-fold cross-validation. Finally, the performance of the SVM classifier was tested on the testing dataset, and the accuracy and area under the receiver operating characteristic curve (AUC) with specificity and sensitivity were reported. Accuracy was computed using the formula (TP + TN) / (TP + TN + FP + FN), where true positives (TP) represent correct predictions of "SP," False Positives (FP) signify cases where "SP" was incorrectly predicted as "FP," False Negatives (FN) denote instances where "FP" was inaccurately predicted as "SP," and True Negatives (TN) indicate accurate predictions of "FP." Additionally, sensitivity was calculated as TP / (TP + FN) and specificity as TN / (TN + FP).

## Results

Clinical and demographic data were as displayed in Table 1, with no significant differences between PD and HC at baseline in age (t = 0.90; *P* = 0.36) or sex distribution (χ^2^ = 0.05; *P* = 0.88). There was a significant increase in total MDS-UPDRS-III (t = 6.11; *P* < 0.001), as well as subscores such as rigidity (t = 4.20; *P* < 0.001), bradykinesia (t = 4.92; *P* < 0.001), gait and postural instability (t = 4.05; *P* < 0.001), and resting tremor (t = 3.75; *P* < 0.001) after 48 months compared to the baseline in PD. Postural or kinetic tremor did not show any significant changes (t = 0.04; *P* = 0.96).

**Table 1:** Clinical and demographic data of the participants.

(Mean ± SD)	Healthy controls (N = 120)	Patients with PD (N = 88)	
		Baseline	48 months
Age, years	61.46 ± 11.11	60.18 ± 9.38	64.18 ± 9.38
Gender (Male: Female)	80:40	60:28	60:28
MDS-UPDRS-III total	–	19.69 ± 8.79	30.38 ± 13.81
Rigidity	–	4.01 ± 2.76	6.05 ± 3.60
Bradykinesia	–	7.93 ± 4.49	12.11 ± 6.56
Postural instability and gait	–	0.66 ± 0.72	1.48 ± 1.75
Postural or Kinetic tremor	–	1.59 ± 1.47	1.60 ± 1.81
Resting tremor	–	2.16 ± 2.07	3.65 ± 3.08

### Group-level gray matter volumetric changes associated with motor symptoms

We did not observe any significant correlations between harmonized GMV_N_ of motor-specific ROIs and total MDS-UPDRS-III or subscores (bradykinesia, rigidity, gait and postural disturbances, resting or postural-kinetic tremor).

### Prediction of motor symptom severity using patient-specific multivariate gray matter volumetric distance

In the multiple linear regression analysis, we included baseline M_GMV_ as a predictor controlled for age and gender, with ∆ (MDS-UPDRS-III) as the outcome variable. In 88 patients with PD, M_GMV_ at baseline significantly predicted the rate of progression of motor symptoms (β (95% CI) = 5.10 (3.52 to 6.69), adjusted R^2^ = 0.33, *P* < 0.0001). Our result implies that a one-unit increase in M_GMV_ at baseline reflects a corresponding change in the motor outcome, on average, by 5.10 points in 48 months. We also predicted the changes in subscores of MDS-UPDRS-III scores, namely., rigidity, bradykinesia, postural instability and gait, postural-kinetic tremor, and resting tremor. The results are shown in Table 2. The analysis revealed significant predictions for changes in rigidity, bradykinesia, gait, and postural instability (*P* < 0.008). Additionally, we observed a borderline level of significance for resting tremor (*P* = 0.03), while postural-kinetic tremor did not show a significant effect (*P* > 0.05).

**Table 2:** Linear regression model of M_GMV_ predicting changes in motor scores.

Changes in motor scores (∆)	β (95% CI)	R^2^	*P*	Cohen’s f^2^	Effect size
MDS-UPDRS-III total	5.10 (3.52–6.69)	0.33	< 0.0001	0.49	Large
Rigidity	0.85 (0.34–1.36)	0.11	0.005	0.12	Small
Bradykinesia	2.26 (1.47–3.04)	0.26	< 0.0001	0.35	Large
Postural instability and gait	0.46 (0.23–0.68)	0.18	0.0001	0.22	Medium
Postural-kinetic Tremor	− 0.11 (− 0.43–0.22)	− 0.02	0.72	–	–
Resting Tremor	0.55 (0.15–0.95)	0.07	0.03	0.08	Small

### Classification of patients based on their motor severity using SVM classifier

Given the finding that M_GMV_ at baseline could predict motor severity, specifically, ∆(MDS-UPDRS-III) total with a large effect size (Cohen’s f^2^ = 0.49) in patients with PD, we next sought to examine if the patients could be differentiated as SP and FP using machine learning. A SVM classifier was tested using M_GMV_, age, sex, and MDS-UPDRS-III at baseline as features with these 88 patients as train data, comprising 46 SP and 42 FP. The clinical and demographic characteristics of patients with PD identified as SP and FP for building the SVM classifier are shown in Table 3.

**Table 3:** Demographic, and clinical characteristics of patients with Parkinson’s disease identified as slower progressors and faster progressors for building the support vector machine classifier.

	Slower progressors (N = 46)	Faster progressors (N = 42)	*p*/χ^2^
Age	59.75 ± 9.05	60.43 ± 9.81	0.66
Sex (male: female)	27: 19	33: 9	0.06
MDS-UPDRS-III at baseline	22.41 ± 9.04	16.71 ± 7.55	0.002
MDS-UPDRS-III at 48 months	24.32 ± 10.68	37.00 ± 13.91	< 0.0001

**Figure 1:**
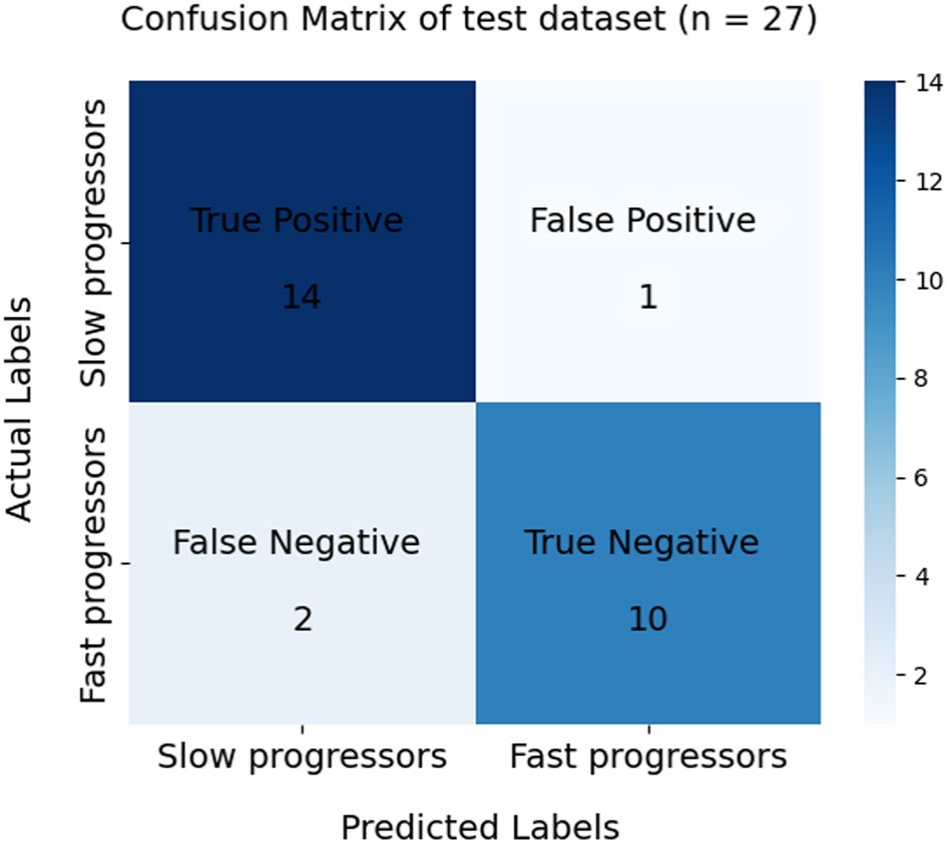
Confusion matrix depicting the performance of an SVM classifier trained to identify slower and faster progressors in test data (n = 27). True positives represent correct predictions of "slow progressors." False positives represent cases where "slow progressors" were incorrectly predicted as "fast progressors." False negatives denote instances where "fast progressors" were inaccurately predicted as "slow progressors." True negatives indicate accurate predictions of "fast progressors".

The accuracy of the SVM classifier for the classification of PD patients as SP and FP for the test data was 89% when M_GMV_, age, gender, and MDS-UPDRS-III at baseline were used as features. The AUC value reached 0.85 with a sensitivity and specificity of 87.50% and 90.91%, respectively. The confusion matrix (summarizing the prediction results) on the test data is shown in Fig. 1. A significant decrease in the model's performance was observed when either M_GMV_ alone or clinical data alone (including age, gender, and MDS-UPDRS-III at baseline) were employed as features, as illustrated in Table 4.

**Table 4:** Performance of a support vector machine classifier with different sets of features as input.

Features	Accuracy	Sensitivity (%)	Specificity (%)	AUC
M_GMV_, Age, Sex, MDS-UPDRS-III at baseline	89	87.50	90.91	0.85
M_GMV_	67	80.00	58.82	0.63
Age, Sex, MDS-UPDRS-III at baseline	70	76.92	64.28	0.71

## Discussion

In the present study, we first demonstrated that group-level GMV reductions at baseline were not associated with changes in motor scores. Second, we introduced a patient-specific summary score capturing the GMV heterogeneity of 40 motor-specific GMV into a single score using MD (M_GMV_) that significantly predicted the rate of progression of motor symptoms in patients with PD. Finally, we classified the patients as SP and FP with higher accuracy using the SVM classifier. Thus, we developed a promising structural MRI-based biomarker for predicting the rate of progression of motor symptoms and classifying patients based on motor symptom severity.

The underlying pathology of PD involves degeneration of dopaminergic neurons in the substantia nigra, neuroinflammation, and the accumulation of misfolded α-synuclein proteins as Lewy bodies and neurites in multiple brain regions^9,28,29^. These processes can result in the alteration of brain morphology and functions. When we conducted group-level volumetric analysis, we found no evidence of a connection between GMV and the overall as well as subscores of motor symptoms as assessed by the MDS-UPDRS-III in patients with PD. Previous studies on the relationship between GMV and motor symptoms yielded contradictory results^12–14,30^. For example, Li et al. reported a negative association between rigidity and the striatum, as well as an inverse relationship between axial symptoms and the left precentral cortex ^14,30^, while others did not observe such correlations^12,31^. These inconsistencies could be due to the variability in the methodology, the statistical evaluation, or the heterogeneity of the patient group. If there is a high level of inter-subject variability (i.e., different brain regions are involved in the different PD patients), then the group-level analyses fail to detect the overall effect for each brain region, which can hinder our understanding of the pivotal role of the brain region(s) in PD pathogenesis. Therefore, identification of a novel metric that can capture the subtle changes in the brain regions in individual patients is necessary to understand the pathology.

Here we present a patient-specific summary score of GMV heterogeneity in individual patients using Mahalanobis distance (M_GMV_). MD is a promising multivariate statistical approach that combines information from numerous brain regions into a single measure, and its application has been successful in traumatic brain injury, epilepsy, and autism^32–36^. M_GMV_ describes the individual GMV heterogeneity by comparing one patient against a group of healthy controls. Hence, the larger the distance, the farther the patient is from the healthy control distribution. A novel finding of this study was that M_GMV_ at baseline significantly predicted the rate of progression of motor symptoms measured using the MDS-UPDRS-III. Specifically, a higher M_GMV_ (reflecting greater gray matter atrophy in the motor-relevant ROIs) was associated with more severe motor symptoms. Furthermore, it also predicted changes in the cardinal motor symptoms of PD, such as rigidity, bradykinesia, and postural instability and gait. M_GMV_ also showed borderline significance for resting tremor (*P* = 0.03) but not for postural-kinetic tremor. PD tremor occurs most frequently at rest, and to a lesser degree with posture and action whereas postural-kinetic predominant tremor is a distinguishing feature of other tremor disorders such as, essential tremor (ET)^37^. Both ET and PD tremor are associated with tremulous activity in the cerebello-thalamo-cortical circuit, but with distinct pathophysiological mechanisms^38^. Specifically, striatal dopaminergic depletion triggers the increased activity in the cerebello-thalamo-cortical circuit in PD tremor, whereas in ET, GABAergic dysfunction in the cerebellar dentate nucleus and brain stem triggers the tremulous activity in the circuit^39^. To the best of our knowledge, this is the first study to report the predictions of motor outcomes using M_GMV_. However, given the borderline significance observed in the prediction of resting tremor, it is essential to interpret this preliminary finding cautiously and seek replication in future studies before drawing definitive conclusions. We hope that we provided a starting point to consider M_GMV_ as a candidate imaging prognostic biomarker for PD, and in the future, it would be interesting to explore its prognostic value in other movement disorders.

Predicting whether a patient is likely to have less severe or more severe motor symptoms can revolutionize clinical decision-making. We utilized SVM classifier to discriminate between patients with SP and FP of motor symptoms. The discriminating ability of our SVM classifier to correctly identify patients as SP and FP was confirmed with an accuracy of 89% and an excellent AUC of 0.85 using M_GMV_ and clinical data as input features. With the higher accuracy of our classifier on the test data, our models’ ability to accurately stratify patients according to their motor outcomes is very promising, and it may act as a useful prognostic classifier in clinics.

However, an essential insight emerged when we used either M_GMV_ alone or clinical data alone as features in our analysis, leading to a significant reduction in the classifier’s accuracy. Specifically, in the case of M_GMV_, the accuracy dropped from 89 to 67%, while for clinical data, it decreased to 70%. These initial findings underscore the crucial importance of integrating clinical variables with M_GMV_. This fusion not only improved model performance but also enabled more accurate predictions to be made.

Our study has a few limitations. First, PD exhibits both motor and non-motor symptoms^40^. The present study focused on the utility of M_GMV_ derived from motor-specific ROIs in predicting and classifying patients based on motor symptoms. In the future, we will interrogate the capability of the multivariate method (MD) to predict non-motor symptoms using ROIs specific to each non-motor symptom. Second, as mentioned in the methodology, there is no standard criteria for classifying SP or FP in the PD literature. Therefore, we followed the most common approach, “median splits”^41,42^ to categorize these patients as SP and FP for developing a machine learning classifier. Even though median splits could be used as a separation point, the marginal samples close to the median may fall in the incorrect group. An alternative solution was to select the first and third quartiles; however, this could significantly lower our small sample size (from n = 88 to n = 47) for classification and could lead to potentially spurious results^43^. Therefore, our SVM classifier results warrant further evaluation in a larger sample. Third, the present study evaluated the clinical utility of M_GMV_ in predicting relatively long-term changes in motor symptoms derived from two time points (baseline and at 48 months). Continuing work to evaluate the clinical utility of M_GMV_ in predicting motor outcomes in prospective and longitudinal data will be necessary to determine its effectiveness. Lastly, MDS-UPDRS-III scores were used for measuring the severity of motor symptoms. These scores are subjective and are prone to a considerable amount of inter-subject variability^44,45^. In the future, reliable scores that provide more precise measurements^46–48^ of the motor symptoms must be tested to improve the clinical relevance.

In summary, we developed a structural MRI-based biomarker to predict the rate of progression of motor symptoms and classify patients based on motor symptom severity. Our findings are an important step towards implementing a novel imaging biomarker for the personalized treatment of Parkinson’s disease. Future research should focus on the reproducibility of this imaging biomarker to ascertain its effectiveness in clinical practice.

### Supplementary Information

Supplementary Information.

## Data Availability

Data used for this manuscript were obtained from the Parkinson’s Progression Markers Initiative database (https://www.ppmi-info.org/access-data-specimens/download-data), RRID:SCR_006431. The datasets that were utilized and/or analyzed during the present study can be made available from the corresponding author upon a reasonable request.

## Code availability

The codes that support the findings of the study are available from the corresponding author upon a reasonable request.
