# Author Correction: MRI-based multivariate gray matter volumetric distance for predicting motor symptom progression in Parkinson's disease

**DOI:** 10.1038/s41598-023-46992-2

**Published:** 2023-11-09

**Authors:** Anupa A. Vijayakumari, Hubert H. Fernandez, Benjamin L. Walter

**Affiliations:** Center for Neurological Restoration, Cleveland Clinic, 9500 Euclid Avenue, Mail Code: S20, Cleveland, OH 44195 USA

Correction to: *Scientific Reports* 10.1038/s41598-023-44322-0, published online 17 October 2023

The original version of this Article contained an error in the Abstract.

“Group-level volumetric analyses revealed that the volumetric reductions in the left striatum were associated with the decline in motor functioning.”

now reads:

“Group-level volumetric analyses at baseline were not associated with the decline in motor functioning.”

The original Article has been corrected.

