# Supplementary material for: MRI-based multivariate gray matter volumetric distance for predicting motor symptom progression in Parkinson's disease
Source: Sci Rep. 2023 Oct 17;13:17704. doi: 10.1038/s41598-023-44322-0 (PMC10582255; doi:10.1038/s41598-023-44322-0)
Supplement: Supplementary file 1 — Supplementary Information. [file 41598_2023_44322_MOESM1_ESM.docx]

**Supplementary Figure S1.** Flowchart of patient selection from the PPMI database. The dark boxes represent the excluded patients.


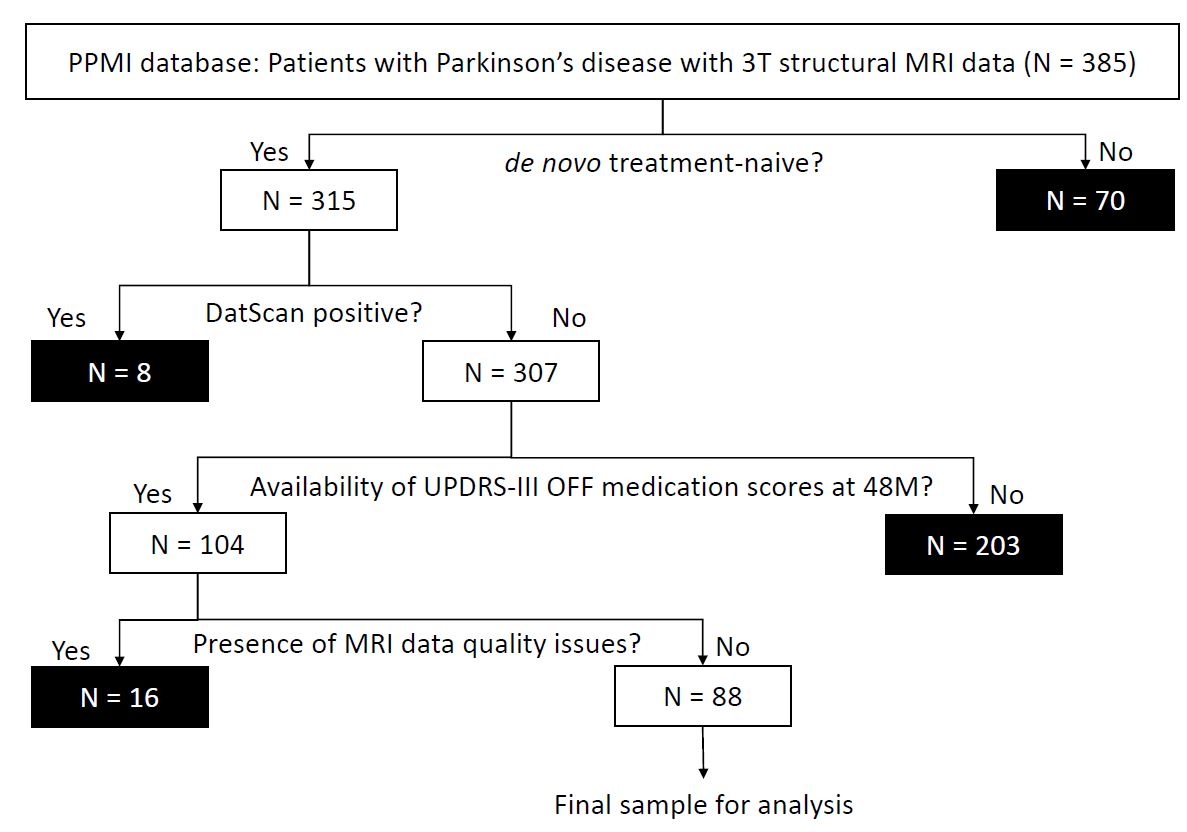


**Supplementary Table S1.** Description of scanning parameters and the number of participants from each scanner.

| Healthy controls (N) | Patients with PD (N) | Scanner | 3T Model | Flip angle,° | Matrix | ST, mm | TE, ms | TI,ms | TR, ms |
| --- | --- | --- | --- | --- | --- | --- | --- | --- | --- |
| 14 | 3 | Siemens | Verio | 9 | 256x256x192 | 1 | 2.7 | 900 | 2300 |
| 77 | 74 | Siemens | TrioTim | 9 | 256x256x176 | 1 | 3 | 900 | 2300 |
| 10 | 6 | GE | Signa HDxt | 13 | 256x256x152 | 1.2 | 3.6 | 450 | 9.1 |
| 5 | - | Philips | Ingenia | 9 | 256x256x192 | 1 | 3.9 | 1 | 6.5 |
| 14 | 5 | Philips | Achieva | 9 | 256x256x192 | 1 | 2.9 | 0 | 6.9 |

*N = number of subjects, ST = slice thickness, TE = echo time, TI = inversion time, TR = repletion time, ms = milliseconds, ° in degrees

**Supplementary Table S2.** 40 brain regions of interest (ROI) from AAL atlas.

| **Sl. No.** | **Brain regions** |
| --- | --- |
| 1 | Left Precentral gyrus |
| 2 | Right Precentral gyrus |
| 3 | Left Supplementary motor area |
| 4 | Right Supplementary motor area |
| 5 | Left Postcentral gyrus |
| 6 | Right Postcentral gyrus |
| 7 | Left Caudate nucleus |
| 8 | Right Caudate nucleus |
| 9 | Left Lenticular nucleus-Putamen |
| 10 | Right Lenticular nucleus-Putamen |
| 11 | Left Lenticular nucleus-Pallidum |
| 12 | Right Lenticular nucleus-Pallidum |
| 13 | Left Thalamus |
| 14 | Right Thalamus |
| 15 | Left Crus I of cerebellar hemisphere |
| 16 | Right Crus I of cerebellar hemisphere |
| 17 | Left Crus II of cerebellar hemisphere |
| 18 | Right Crus II of cerebellar hemisphere |
| 19 | Left Lobule III of cerebellar hemisphere |
| 20 | Right Lobule III of cerebellar hemisphere |
| 21 | Left Lobule IV-V of cerebellar hemisphere |
| 22 | Right Lobule IV-V of cerebellar hemisphere |
| 23 | Left Lobule VI of cerebellar hemisphere |
| 24 | Right Lobule VI of cerebellar hemisphere |
| 25 | Left Lobule VIIB of cerebellar hemisphere |
| 26 | Right Lobule VIIB of cerebellar hemisphere |
| 27 | Left Lobule VIII of cerebellar hemisphere |
| 28 | Right Lobule VIII of cerebellar hemisphere |
| 29 | Left Lobule IX of cerebellar hemisphere |
| 30 | Right Lobule IX of cerebellar hemisphere |
| 31 | Left Lobule X of cerebellar hemisphere |
| 32 | Right Lobule X of cerebellar hemisphere |
| 33 | Lobule I-II of vermis |
| 34 | Lobule III of vermis |
| 35 | Lobule IV-V of vermis |
| 36 | Lobule VI of vermis |
| 37 | Lobule VII of vermis |
| 38 | Lobule VIII of vermis |
| 39 | Lobule IX of vermis |
| 40 | Lobule X of vermis |
